# Harnessing Shared Identities to Mobilize Resilient Responses to the COVID‐19 Pandemic

**DOI:** 10.1111/pops.12726

**Published:** 2021-02-19

**Authors:** Vivian L. Vignoles, Zahira Jaser, Frankiebo Taylor, Evangelos Ntontis

**Affiliations:** ^1^ University of Sussex; ^2^ Canterbury Christ Church University

**Keywords:** COVID‐19, identity entrepreneurship, national identity, political leadership, public health

## Abstract

Shared social identifications (family, community, nation, humanity) predict normative actions and psychological well‐being and can be invoked discursively by leaders to mobilize their followers. We illustrate the potential for harnessing shared identities to mobilize resilient public responses against COVID‐19. Study 1 explored which patterns of social identification predicted protective behaviors (personal hygiene, physical distancing), prosocial actions (helping proximal and distal others), and psychological well‐being (mental well‐being, depressive symptoms, anxiety) among 560 U.K. adults surveyed during lockdown. Study 2 contrasted Prime Minister Ardern's use of identity‐based rhetoric to mobilize New Zealanders, with Prime Minister Johnson's use of individualistic appeals to the U.K. public. Our findings suggest how political leaders might beneficially use social identities in communications about extreme events.

**Highlights**

- Political leaders seeking to mobilize cooperative, resilient public responses to the COVID‐19 pandemic should portray themselves and their followers as sharing multiple group identities.
- They should attribute collective agency and norms of solidarity to these groups.
- They should portray desired public behaviors as morally necessary acts of mutual helping, stemming from the “character” of the invoked group identities.
- They should show they have a personal stake in the political choices they make, as members of the groups affected.

During a global pandemic, a crucial task for political leaders is mobilizing the public rapidly to engage in novel collective behaviors (e.g., hand sanitizing, mask wearing) and relinquish existing potentially harmful behaviors (e.g., face‐to‐face meetings, physical greetings). Achieving large‐scale behavior change is difficult because it requires appealing simultaneously to individuals with very different values, concerns, and interests. To bridge these differences, behavioral scientists propose that leaders must cultivate and harness a sense of shared identity among the public (Van Bavel, Baicker, et al., 2020). Here, we report findings of two studies illustrating how social identification can predict resilient public responses to COVID‐19 (Study 1) and how political leaders can rhetorically facilitate these social identity processes among their followers (Study 2).

## SHARED IDENTITIES AND RESILIENCE

Shared identities offer at least three potential benefits for societal resilience in a pandemic: motivating action, fostering well‐being, and enabling leadership.

### Motivating Action

When a social group/category is contextually salient, people tend to see themselves as group members and act according to group norms (Abrams, Wetherell, Cochrane, Hogg, & Turner, 1990; Hogg & Turner, 1987). Thus, a sense of shared identity with others—*social identification*—is an important basis for collective action on behalf of one's group (van Zomeren, 2013), including mutual helping in emergencies (Drury, Carter, Cocking, Ntontis, & Guven, 2019). Individuals may underestimate their personal susceptibility to COVID‐19 infection, and most infected individuals have mild or no symptoms, but infecting others could be fatal (Kuper‐Smith, Doppelhofer, Oganian, Rosenblau, & Korn, 2020). Evidence suggests people who are overconfident about their personal immunity engage in better hand hygiene when reminded of consequences for others (Grant & Hofmann, 2011). Hence, protective measures such as handwashing and physical distancing can be viewed as collective acts of solidarity. Social identification may motivate compliance with these measures, as well as prosocial actions such as shopping for others or donations.

### Fostering Well‐Being

Social identification with a positively valued group can provide feelings of belonging and support, boosting health and well‐being (Haslam, Jetten, Postmes, & Haslam, 2009; Jetten et al., 2017) and reducing negative psychological states such as depression (Cruwys et al., 2013) or low self‐esteem (Schmitt, Spears, & Branscombe, 2003). During the 2015 MERS‐CoV outbreak, dialysis patients experienced significantly higher stress after two weeks' isolation (Kim et al., 2019). Health‐care professionals and medical staff risk developing psychiatric disorders (such as anxiety disorders and PTSD) following infectious disease outbreaks (Park, Lee, Park, & Choi, 2018). Yet, group identification can reduce stress linked to severe illnesses as well as occupational strain (Haslam, O'Brien, Jetten, Vormedal, & Penna, 2005). Thus, shared identities may function as a *social cure* (Jetten et al., 2017), helping people cope with the uncertainty and strains of a pandemic.

### Enabling Leadership

Persuading the public to act collectively against a global pandemic requires effective leadership. Recent advances view effective leadership as a relational process between leaders and their followers based on shared social identity (Haslam, Reicher, & Platow, 2020). Language plays a key role in identity‐based leadership, reflected in analyses focusing on the rhetorical and interactional nature of social categories in talk (Housley & Fitzgerald, 2015). *Identity entrepreneurs* rhetorically construct their audiences by invoking particular social categories and defining their boundaries, norms, and interests (Reicher & Hopkins, 2001), sometimes with major societal consequences (Reicher, Cassidy, Wolpert, Hopkins, & Levine, 2006). Leaders establish the collective social subject to be mobilized (Fladerer, Steffens, & Haslam, 2020) and define the nature of particular events (e.g., the COVID‐19 pandemic) and of the desired collective responses (Reicher & Hopkins, 1996). They also construct and position themselves as prototypical group members (Haslam et al., 2020), representing and embodying the values of their socially diverse audiences (Augoustinos & De Garis, 2012). Constructing a shared social identity with one's audience facilitates trust in one's leadership and secures compliance with norms defined by the leader—identity‐based followership (Steffens, 2020). Thus, identity‐based leadership can facilitate social identification, motivate group‐based action, and foster well‐being, which may be crucial for promoting resilient collective responses to COVID‐19.

## CURRENT STUDIES

We report two studies investigating how these benefits of shared identities might be harnessed to mobilize resilient responses to the COVID‐19 pandemic. Study 1, an online survey, explored the role of social identification with family, local community, nation, and all humanity in predicting compliance with protective measures, prosocial actions, and psychological well‐being among U.K. adults during the first phase of lockdown. Study 2, a discursive analysis, explored the use of identity‐based rhetoric by two contrasting political leaders—U.K. Prime Minister Boris Johnson and New Zealand Prime Minister Jacinda Ardern—seeking to mobilize public responses to COVID‐19.

## STUDY 1

For Study 1, we examined which patterns of social identification predicted individual differences in protective behaviors, prosocial actions, and psychological well‐being among U.K. adults during the 4th to 7th weeks of lockdown. We focused on four identity categories that might be salient in the context of COVID‐19: humanity (because the pandemic is global), the nation (because protective measures were decided and promoted nationally), local community (because geographical proximity enables transmission and helping), and family (because families often connect members of lower‐risk and higher‐risk age groups).

### Method

Between April 17 and May 9, 2020, 560 adults (458 female, 95 male, 3 nonbinary/queer, 4 unspecified) aged 18 to 85 (mean = 43.36, *SD* = 14.67), residing across the four U.K. nations, completed our online survey. The survey comprised demographic questions (including impacts of lockdown on work), brief measures of well‐being (mental well‐being, depression, anxiety), perceived severity of the pandemic, own and close others' suspected coronavirus symptoms, social identification with family, community, nation, and humanity, and self‐reported protective and prosocial actions (personal hygiene, physical distancing, helping proximal and distal others). Participants, procedure, measures, and analyses are described fully in the online supporting information and Supplementary Tables S1–S5.

### Results and Discussion

Social identification measures were associated with protective and prosocial actions, well‐being, and greater perceived severity (Table S3). Controlling for age, gender, nation of residence, and experiences of COVID‐19 (own and close others' symptoms, impact on work, days into lockdown), social identification measures predicted significant and substantial additional variance (Δ*R^2^*) in all outcomes except personal hygiene (Tables S4 and S5). Thus, social identification can predict desired outcomes during a global pandemic.

**Figure 1:**
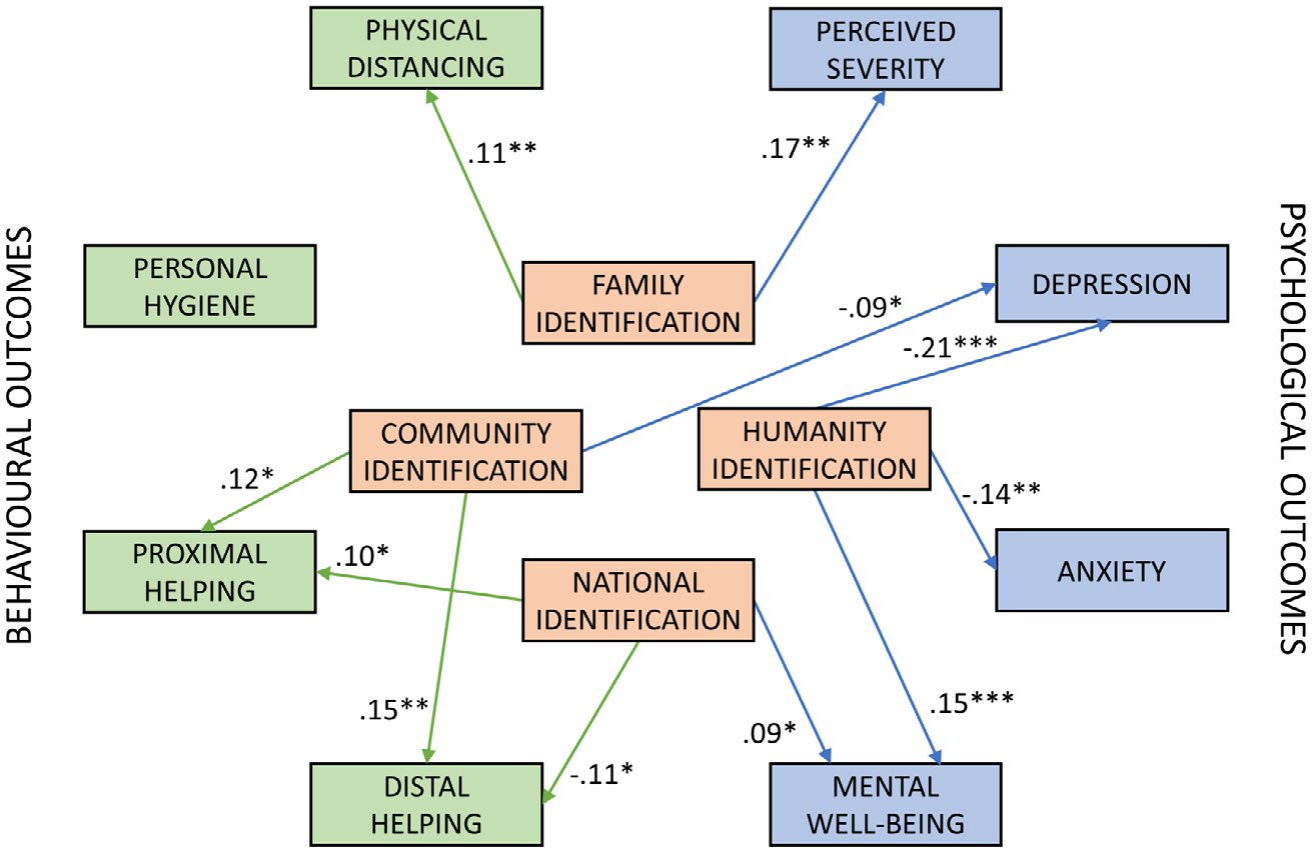
Statistically significant paths from social identification to outcomes. Numbers are standardized regression weights (*β*) controlling for age, gender, nation of residence, and experiences of pandemic. See Tables S4 and S5 in the online supporting information for full results with unstandardized weights and bootstrapped confidence intervals. ***p < .001, **p < .01, *p < .05.

Crucially, each social identification measure uniquely predicted different outcomes. Figure 1 summarizes regression paths involving social identification that reached statistical significance. These findings are culturally and historically contingent and may differ where the content and norms of identity categories are constructed differently. Family identification predicted physical distancing (and perceived severity). This is striking, as compliance with physical distancing was extremely high at that time, leaving little room for variation (Table S2). Community identification predicted helping proximal and distal others (and lower depressive symptoms) (for converging findings, see Stevenson, Wakefield, Drury, & Felsner, 2020). Humanity identification predicted mental well‐being, and lower anxiety and depressive symptoms, unlike pre‐pandemic findings where less inclusive identities better predicted well‐being (Hodges & Gore, 2019). Thus, in the early‐lockdown United Kingdom, family and community identities respectively showed strongest links to protective and prosocial actions, whereas humanity identity showed strongest evidence of social cure effects.

National identification did not significantly predict protective actions. It predicted somewhat higher mental well‐being and showed complex relations with prosocial actions—predicting more actions helping proximal others, but *fewer* actions helping distal others. We explored the latter finding in follow‐up analyses separating three facets of social identification (see online supporting information). Participants higher on national *satisfaction* (i.e., proud to be British)—rather than national *solidarity* (i.e., committed to British people) or national *centrality* (i.e., British is an important part of identity)—reported lower distal helping. Unwillingness to help distal others might be linked to national hubris, rather than national identification per se. Still, the failure of British national identification to predict desired actions is striking, raising questions about how the content and norms of British national identity were constructed at the time.

## STUDY 2

Hence, in Study 2, we compared the use of identity‐based rhetoric by U.K. Prime Minister Boris Johnson and New Zealand Prime Minister Jacinda Ardern at the onset of the pandemic. Although affluent and culturally similar island nations, the United Kingdom and New Zealand showed diverging early trajectories for the pandemic. Up to May 29, 2020, England had the highest cumulative excess mortality rate of 23 European countries, with Scotland 3rd, Wales 5th, and Northern Ireland 8th (Office for National Statistics, 2020), whereas New Zealand almost entirely eliminated the virus during the same period (Baker, Wilson, & Anglemyer, 2020). Johnson and Ardern have been extensively compared by political commentators on COVID‐19 (e.g., The National, 2020). Ardern's leadership has been hailed as exemplary, but not yet analyzed from a social identity perspective (Wilson, 2020). We analyzed the first 10 speeches/briefings regarding COVID‐19 by each leader. We explored how each invoked specific social categories and how they rhetorically constructed themselves and their audiences, when requesting adherence to protection measures.

### Method

Official transcripts were sourced from the respective governments' websites. We adopted a discursive thematic analysis (Clarke, Braun, & Hayfield, 2015), informed by the rhetorical shift in the social identity approach (Reicher & Hopkins, 2001) and the ethnomethodological approach to category use (Housley & Fitzgerald, 2015). Thus, we treated identity as accomplished through rhetorical practices, exploring variability in category use and content, positioning of the self and of the respective audiences, and the social action performed by invoking particular categories. We used an iterative approach cycling from data to theory, back to data, identifying theoretical codes and progressively forming wider themes. Three authors worked on the analysis. Verbatim quotations illustrate our analysis. For further details of the data corpus and analyses, and for additional quotations, see the online supporting information and Supplementary Table S6.

### Findings

We identified two subthemes illustrating how the leaders positioned themselves and their audience in requests for action, and two subthemes highlighting how they rhetorically constructed their audiences, by invoking social identities and associated norms. To respond to Study 1 findings, we pay special attention to the interactional work accomplished in uses and constructions of national categories.

#### Leader Self‐Positioning in Requests for Action

##### Positioning Self and Audience

We found striking differences in how the leaders positioned themselves in relation to their audiences when requesting protective actions:

*Johnson*: I want to repeat that everyone—everyone—must follow the advice to protect themselves and their families, but also—more importantly—to protect the wider public.

*Ardern*
*:*
That means we need friends, family and neighbours to support our older New Zealanders and people who may be in this group by doing simple things like keeping in contact and dropping off food or other supplies.

Here and in further quotes (Table S6), Johnson constructs his audience in generic terms (“everyone,” “wider public,” “people”), whereas Ardern positions her audience as an inclusive national category (“we as a nation,” “our older New Zealanders”) while simultaneously invoking further commonplace categories associated the provision of social support (“friends,” “family,” “neighbours”). Ardern uses the first‐person plural to position herself and her government within the national category, framing her requests as representing the collective interest (“we want to make sure,” “we need to”). In contrast, Johnson's use of diverging pronouns creates distance between himself as the leader of the government (“I want,” “I know”) and his audience (“you,” “them,” “themselves”).

##### Framing Government Decisions

The two leaders also differently portrayed governmental decisions. Ardern frames her decisions as *moral imperatives* (“New Zealanders' public health comes first”, “The worst‐case scenario is simply intolerable”: Table S6), in which she has a personal stake:

*Ardern*: For those of you who are parents or caregivers, you will have questions about schools and education facilities. . . . I can assure you we are constantly monitoring these settings to keep children safe. As a mum, I can assure you that is my key consideration.

Whilst positioning herself as both maker and recipient of government decisions through employing commonplace categories that can facilitate rapport with a diverse national audience (“as a mum”), Ardern also attributes agency to her listeners (“you will have questions”).

Johnson frames his decisions as *technical requirements* (“we need to keep people apart,” “we need health workers who are also parents to continue to go to work”: Table S6). His language seemingly locates agency with the government rather than the public:

*Johnson*: And even if you don't have symptoms and if no one in your household has symptoms, there is more that we need you to do now.

By separating “we” (decision‐makers) from “you” (recipients of instructions), it is unclear whether the decision‐makers are also expected to follow their own instructions.

###### Constructing the National Group and Defining Its Content, Norms, and Interests

####### Invoking Collective Resilience

Both leaders constructed the pandemic as a national problem that could be overcome through collective agency (Johnson: “We will beat the coronavirus and we will beat it together”; Ardern: “let's finish what we started”). Typical of national mobilization rhetoric (see Reicher & Hopkins, 2001), both invoked explicit or implicit wartime references to characterize the nation as resilient to adversity (Ardern: “ANZAC weekend,” “the amazing sacrifice of our forebears,” “a very different battle”; Johnson: “every one of us is directly enlisted,” “as they have in the past so many times”: Table S6 in the online supporting information).

######## Framing Protective Actions

However, the two leaders constructed very different rationales for adopting protective measures:

*Johnson*: Some people may of course be tempted to go out tonight. But please don't. You may think you are invincible, but there is no guarantee you will get mild symptoms, and you can still be a carrier of the disease and pass it on to others. So that's why, as far as possible, we want you to stay at home, that's how we can protect our NHS and save lives.

*Ardern*: In the face of the greatest threat to human health we have seen in over a century, Kiwis have quietly and collectively implemented a nationwide wall of defence. You are breaking the chain of transmission. And you did it for each other. As a Government, we may have had pandemic notices. We may have had powers that come with being in a national emergency. But you held the greatest power of all. You made the decision that together, we could protect one other. And you have.

Johnson appeals to listeners primarily as individuals and asks them to protect *others* and generically “save lives,” whereas Ardern stresses the importance of protecting *each other*, emphasizing the *mutuality* of protective actions. Referring only obliquely to national identity by invoking the National Health Service (“protect our NHS”), Johnson emphasizes the frustration that collective compliance might cause (“You might be tempted to go out tonight”), attributes limited agency to the public (“You may think you are invincible, but …”), and invokes modest collective goals (“slow the spread”). In contrast, Ardern specifies a shared national mentality (“how we must all collectively think”), links the national category to smaller commonplace social categories consonant with her audience (family, friends, neighbors), and defines mutual helping as a central characteristic of national identity (“putting each other first . . . is what we as a nation do so well.”: Table S6 in the online supporting information). Moreover, Ardern attributes progress to collective national agency (“Kiwis have quietly and collectively implemented a nationwide wall of defence,” “you held the greatest power of all”) and aspires to “breaking the chain of transmission.”

#### Discussion

We found notable differences in the leaders' uses of identity‐based rhetoric. Although both made some use of national wartime references, Johnson mainly positioned himself as an individual, constituted a generic audience of “people,” emphasized the benefits of adherence for protecting “others” and national assets (NHS), and portrayed an agentic government separate from a hopefully compliant public. Ardern positioned her audience simultaneously as a national group and as members of the same smaller communities that were associated with protective and prosocial actions in Study 1 (family, neighbors), imbued this national group with collective agency, and effectively construed group norms of mutual helping and solidarity. Ardern's rhetoric matches recommendations for facilitating adherence to health protection measures, including clear guidance, behavioral norms that emphasize collective well‐being and protection, and a sense of togetherness (Drury et al., 2019). Novel insights regarding identity‐based leadership (Haslam et al., 2020) concern how Ardern positioned herself as having a *shared stake* with her audience in the decisions made, constructed her decisions as *moral imperatives*, and attributed *collective agency* to the nation to address the pandemic through *mutual solidarity*.

### GENERAL DISCUSSION

These studies illustrate the potential of shared social identities for translating political decisions into public action during a global pandemic. The design of these studies does not permit strong causal inferences, but the findings suggest a pathway from political rhetoric to public action via social identification, consistent with research into identity‐based leadership (Haslam et al., 2020). During March 2020, Boris Johnson invoked British national resilience in speeches about COVID‐19 but emphasized the disadvantage for *individuals* in adopting protective measures, avoiding discourses of moral necessity, collective agency, and *mutual* solidarity, as emphasized by Jacinda Ardern. During April–May 2020 in the United Kingdom, family identification predicted protective action, community identification predicted helping, and humanity identification predicted well‐being; but national identification did not predict protective actions and showed ambivalent associations with helping. Intriguingly, initial results from a 67‐country survey suggest that national identification predicted public health engagement and support very weakly in the United Kingdom, but more strongly in New Zealand (Van Bavel, Cichocka, et al., 2020).

Meanings of national identities are shaped partly by political discourse (Reicher & Hopkins, 2001). Thus, differences in how leaders construct identity categories may help explain why British national identification was not more strongly associated with protective actions, perhaps contributing to understanding why the United Kingdom and New Zealand had such different early trajectories during the pandemic. Had Johnson linked British national identity rhetorically to family and community solidarity and to moral imperatives of mutual helping and protection, we might have expected British national identification to motivate and thus predict desired outcomes. We summarize our recommendations for identity‐based political leadership during COVID‐19 in the Highlights box at the beginning of this article.

## Data Availability Statement

Study materials and data can be found in the OSF repository at http://dx.doi.org/10.17605/OSF.IO/P6KAW.

## Supporting information


**Study 1**. Supplementary Information
**Study 2**. Supplementary Information
**Table S1**. Descriptive Statistics and Rotated Component Loadings for Items Measuring Protective and Prosocial Actions
**Table S2**. Means, Standard Deviations and Zero‐Order Correlations of Main Study 1 Measures
**Table S3**. Bivariate Correlations with 95% Bias‐Corrected Accelerated Confidence Intervals (CI; 10,000 Requested Bootstrap Resamples) Testing the Associations between Social Identification and Outcome Measures
**Table S4**. Multiple Regression Parameters with 95% Bias‐Corrected Accelerated Confidence Intervals (CI; 10,000 Requested Bootstrap Resamples) Testing the Associations between Social Identification and Behavioural Outcome Measures
**Table S5**. Multiple Regression Parameters with 95% Bias‐Corrected Accelerated Confidence Intervals (CI; 10,000 Requested Bootstrap Resamples) Testing the Associations between Social Identification and Psychological Outcome Measures
**Table S6**. Additional Quotations Illustrating Each Discursive Theme
