# Supplementary material for: Harnessing Shared Identities to Mobilize Resilient Responses to the COVID‐19 Pandemic
Source: Polit Psychol. 2021 Feb 19;42(5):817–26. doi: 10.1111/pops.12726 (PMC8013210; doi:10.1111/pops.12726)
Supplement: Supplementary file 1 — Study 1. Supplementary Information Study 2. Supplementary Information Table S1. Descriptive Statistics and Rotated Component Loadings for Items Measuring Protective and Prosocial Actions Table S2. Means, Standard Deviations and Zero‐Order Correlations of Main Study 1 Measures Table S3. Bivariate Correlations with 95% Bias‐Corrected Accelerated Confidence Intervals (CI; 10,000 Requested Bootstrap Resamples) Testing the Associations between Social Identification and Outcome Measures Table S4. Multiple Regression Parameters with 95% Bias‐Corrected Accelerated Confidence Intervals (CI; 10,000 Requested Bootstrap Resamples) Testing the Associations between Social Identification and Behavioural Outcome Measures Table S5. Multiple Regression Parameters with 95% Bias‐Corrected Accelerated Confidence Intervals (CI; 10,000 Requested Bootstrap Resamples) Testing the Associations between Social Identification and Psychological Outcome Measures Table S6. Additional Quotations Illustrating Each Discursive Theme [file POPS-42-817-s001.docx]

# Online Appendices for

# Harnessing shared identities to mobilise resilient responses to the COVID-19 pandemic

# Study 1: Supplementary Information

## Historical Timeline and Procedure

The UK government put lockdown restrictions in place on 23^rd^ March 2020, introducing a three-point message: “Stay at home. Protect the NHS. Save lives”. On 10^th^ May, after seven weeks of lockdown, the UK Prime Minister announced partial easing of restrictions in England and introduced a revised three-point message: “Stay alert. Control the virus. Save lives.” Devolved governments in Scotland, Wales and Northern Ireland did not make parallel changes, nor did they adopt the new three-point message. We launched our online questionnaire on 17^th^ April 2020, four weeks into the initial lockdown period. The questionnaire was finally closed on 20^th^ May 2020, but we decided to use a cut-off data of 9^th^ May for inclusion in our analyses, given the change in government messaging from 10^th^ May and that only 9 participants had completed the study from 10^th^ May onwards.

Study 1 was conducted by FT and VV, with additional input to the selection of measures by EN. Ethical approval was granted by the School of Psychology, University of Sussex (reference: ER/FT83/2). The study was created online using the Qualtrics survey platform. Participants across the UK were recruited using a snowball sampling technique beginning with Facebook posts (including local community and COVID-related pages), Instagram posts, and group e-mails. After reading an information page and confirming their informed consent and that they were over 18 years of age, participants completed six questionnaire sections: (1) demographic questions, (2) well-being measures, (3) experiences of COVID-19 (e.g., own and others’ symptoms), (4) social identification scales, (5) protective and prosocial behaviours, and (6) additional questions on buying groceries and medicines (which were for another project). The debriefing page explained the goals of the study and provided an e-mail address to contact the researchers, as well as links to the NHS coronavirus information page and recommended mental health apps.

## Participants

Between 17^th^ April and 9^th^ May, 738 individuals consented to participate, of whom 560 (75.9%) provided sufficient data for inclusion in our analyses. Participants were aged 18 to 85 years (mean = 43.36, SD = 14.67). A majority (*n* = 458; 81.8%) identified as female, 95 (17.0%) as male, 3 as non-binary/queer, and 4 preferred not to say. Thus, women were overrepresented compared to the UK population (51.1% of UK adults over 18 years in mid-2019 were female: Office for National Statistics, 2020), as is often the case for online surveys (e.g., Busby & Yoshida, 2013; Whitaker et al., 2017). Most (92.9%) reported their ethnicity as White, and most (90.9%) freely described their nationality as British (including UK, English, Scottish, Welsh, Irish, or some combination of these terms); 87.3% reported their ethnicity as White AND their nationality as British, which closely matches an estimated 87.2% of the UK population who were classified as White British in 2014 (Institute of Race Relations, 2020). Although women were overrepresented, the sample was diverse in terms of geographical dispersion, (impact on) work, and experiences of the virus.

**Geographical dispersion.** Participants resided in at least 69 counties across the UK; 492 participants (89.6%) resided in England (across 46 out of 48 English counties), 35 (6.4%) in Scotland (across at least 13 out of 32 Scottish council areas), 16 (2.9%) in Wales (across 6 out of 8 preserved counties of Wales), and 6 (1.1%) in Northern Ireland (across 4 out of 8 Northern Irish counties and county boroughs). Although residents of Scotland, Wales and especially Northern Ireland were underrepresented compared to the latest available UK population statistics (England 84.3%; Scotland 8.2%; Wales 4.7%; Northern Ireland 2.8%; Office for National Statistics, 2020), the sample was geographically dispersed both across and within the four nations of the UK.

**Work and lockdown impacts.** Most participants (77.2%) worked across a wide range of sectors, including education, health and social care, retail and catering, accounting and finance, administration, transport, arts and media, business management, social and youth work, cleaning/housekeeping, and various other roles; a further 8.6% were retired, 8.2% were students, 3.4% described themselves as homemakers or full-time parents, and 2.3% reported their occupation as unemployed, sick or disabled. Participants reported a range of impacts of lockdown on their work: 101 (18.0%) were still mostly working as normal; 166 (29.6%) were working remotely; 78 (13.9%) were temporarily not working (with furlough pay); 9 (1.6%) had lost their jobs; 105 (18.8%) reported “other” impacts on their working life (e.g., reduced hours, changed shift patterns, mixed remote and on-site working); and 101 (18.0%) reported that the question was not applicable (e.g., if they were retired or full-time students).

**Own and other’s symptoms.** We asked participants about their own and close others’ symptoms that “might be due to the coronavirus” (see below for item wordings). Regarding participants’ own symptoms: 347 (62.0%) reported having experienced no symptoms, 133 (23.8%) had experienced mild symptoms (e.g., headache, conjunctivitis, blocked or runny nose, sore throat, diarrhoea), 76 (13.6%) had experienced moderate symptoms (e.g., persistent cough, fever, exhaustion), and 4 (0.7%) had experienced severe symptoms (e.g. pneumonia, oxygen deficiency, symptoms requiring hospitalisation). Regarding close others: 289 (51.7%) knew at least one person who had experienced moderate symptoms, 123 (22%) knew at least one person who had required hospitalisation, and 66 (11.8%) knew at least one person who had died as a result of suspected coronavirus.

## Materials and Measures

### Well-being Measures

**Mental well-being.** Participants completed the Short Warwick Edinburgh Mental Well-being Scale (Tennant et al., 2007). This scale has been shown to be a valid and reliable tool for measuring well-being (Stewart-Brown et al., 2011). Participants responded to seven items (e.g., “I’ve been feeling optimistic about the future”) rating their experiences over the last two weeks, using a 5-point response scale (1 = None of the Time; 5 = All of the Time). The scale showed good internal consistency (𝛼 = .82).

**Anxiety and depression.** Participants completed the Patient Health Questionnaire-4 (PHQ-4: Kroenke et al., 2009), an ultra-brief but reliable screening measure for symptoms of depression and anxiety. Participants responded to two depression items (e.g., “Little interest or pleasure in doing things”) and two anxiety items (e.g., “Feeling nervous, anxious, or on edge”) rating their experiences over the last two weeks on a 4-point response scale (0 = Not at all; 3 = Nearly every day). Spearman-Brown split-half reliability coefficients (Eisinga et al., 2013) showed good internal consistency for anxiety (ρ_S-B_ = .87) and depression (ρ_S-B_ = .81).

### Experiences of the Virus.

**Perceived severity.** Participants responded to a single item, “How serious do you believe this pandemic to be?” on a scale from 0 to 10 (0 = “not at all”; 10 = “extremely”).^[[1]](#footnote-1)^

**Personal symptoms.** Since COVID-19 testing was not widely available in the UK at that time, we asked participants “Have you personally experienced symptoms that you suspect are due to the coronavirus?”. Response options were “I have experienced no symptoms that might be due to the coronavirus” (coded 1), “I have experienced mild symptoms (e.g. headache, conjunctivitis, blocked or runny nose, sore throat, diarrhoea) that might be due to the coronavirus” (coded 2), “I have experienced moderate symptoms (e.g. persistent cough, fever, exhaustion) that might be due to the coronavirus” (coded 3) or “I have experienced severe symptoms (e.g., pneumonia, oxygen deficiency, symptoms requiring hospitalisation) that might be due to the coronavirus” (coded 4).

**Others’ symptoms.** We asked participants three questions about the symptoms of close others: “Has anyone in your close social circle (e.g., family, friends, colleagues) [experienced moderate symptoms of suspected coronavirus? /severe symptoms of suspected coronavirus that required hospitalisation? /died as a result of suspected coronavirus?]” Participants answered yes or no, with a box to report the number of people they knew who had been affected in each way. We did not ask about mild symptoms, as we expected that participants might not be aware if people they knew had experienced mild symptoms. For analyses, we computed a single index, coded 1 if they knew nobody who had experienced moderate symptoms or worse, 2 if they knew at least one person who had experienced moderate symptoms (but none with severe symptoms or who had died), 3 if they knew at least one person who had experienced severe symptoms (but none who had died), and 4 if they knew at least one person who had died as a result of suspected coronavirus.

### Social Identification Measures

Social identification with family, local community, British people and all humanity were measured using parallel versions of Koc’s (2017) 12-item social identification scale. This measure is closely derived from well-used measures of social identification (e.g., Becker & Tausch, 2014; Leach et al., 2008), was validated in a non-student non-Western cultural sample to reduce cultural biases in item selection, and includes three subscales: solidarity, centrality and satisfaction (4 items each). Unlike previous measures, each subscale includes a balance of forward and reverse-coded items, to avoid a potential confound between social identification and acquiescent response style that might otherwise lead to spurious positive correlations with other measures (Podsakoff et al., 2012).

Participants rated items on a 7-point scale (1 = strongly disagree; 7 = strongly agree). Examples are “The fact that I am part of my family is an important part of my identity” (family centrality); “I feel committed to my local community” (local community solidarity); “I am unhappy about being British” (national satisfaction reversed). All four social identification scales showed excellent reliability: *family identification* (α = .90), *community identification* (α = .92), *national identification* (α = .93), *humanity identification* (α = .90). Solidarity, centrality and satisfaction subscales of each measure all showed acceptable reliabilities (all α > .74).

### Protective and Prosocial Actions

An initial pool of 33 items^[[2]](#footnote-2)^ measuring protective and prosocial actions was adapted and extended from Rubin and colleagues’ (2014) measure of behaviours during an influenza pandemic. To measure *protective behaviours*, we supplemented items from Rubin et al. (e.g., “Cleaned or disinfected things you might touch (doorknobs, surfaces) more often than usual”), with additional items reflecting the UK government guidance for reducing the spread of COVID-19 (e.g., “Stayed at least 2 metres at all times from people who are not part of your household”). Moreover, we added items on *prosocial actions*, such as “Donated money to charity or NHS” and “Offered to shop for vulnerable people”. Participants reported whether they had performed each action during the past 7 days due to the COVID-19 pandemic. Possible responses were yes (coded 1), no (coded 0), not sure (coded 0.5) or N/A (treated as missing data).

Note that these items would not be expected to follow classic psychometric principles. Some actions were only applicable to a minority of participants (e.g., those who had children, or used public transport), and some might also be used in substitution for each other (e.g., donating money or donating food). Hence, we did not expect to find internal consistency, nor that the items would necessarily show a ‘clean’ factor structure. We therefore prioritised conceptual coherence over statistical properties when creating our action measures. To gain insight into dimensionality, we ran Categorical Principal Components Analyses treating items as ordinal and using oblimin rotation. We found four interpretable factors, which we named (I) personal hygiene, (II) proximal helping, (III) physical distancing, and (IV) distal helping. Item-level descriptive statistics and rotated component loadings are shown in Table S1. Of the 33 initial items, 20 loaded cleanly (>.4) on their respective components, 12 cross-loading items were attributed to subscales based on conceptual fit (which usually, but not always, coincided with their highest factor loading), and one item was excluded from further analyses (“followed a healthy diet or took vitamin supplements”).

We created four behavioural indices for use in our main analyses, expressed as the percentage of applicable actions in each category that participants reported having taken (or not taken) in the past week. One physical distancing item (“met with people outside your household for non-essential reasons”) was reverse coded. Because these were behavioural composite scores, rather than psychometric measures, internal consistency was expected to be mediocre: personal hygiene (8 items; α = .69), physical distancing (13 items; α = .61), proximal helping (5 items; α = .69), distal helping (6 items; α = .50).

## Main Analyses

Table S2 reports descriptive statistics and bivariate correlations for our main measures. To allow for non-normal distributions, we conducted bootstrapped analyses with 10000 resamples. Correlations between the four social identification measures and eight outcome measures are shown in Table S3.

For our main analyses, we conducted bootstrapped hierarchical regression analyses. For each outcome, Model 1 included age, gender, nation of residence, and experiences of COVID-19 (own and close others’ symptoms, impact on work, and days into lockdown); Model 2 added social identification measures. Full results of these models are shown in Tables S4 and S5. Significant standardised paths are summarised in Figure 1.

## Supplementary Results

An unexpected finding was that national identification negatively predicted helping distal others. To explore this finding further, we re-ran our analyses replacing the national identification scale with its three subscales: solidarity, centrality and satisfaction. Distal helping showed a significant negative correlation with national satisfaction (*r* = -.09, *p* = .04, 95% CI: -.17, -.004), whereas correlations with national solidarity (*r* = -.02, *p* = .58, 95% CI: -.10, .06), and national centrality (*r* = -.08, *p* = .07, 95% CI: -.15, .003) did not reach significance. In our weighted bootstrapped regression model, national satisfaction was a marginally significant negative predictor of distal helping (β = -.15; B = -2.29, *p* = .05, 95% CI: -4.62, 0.02). However, distal helping was not significantly predicted by national solidarity (β = .06; B = 1.02, *p* = .44, 95% CI: -1.48, 3.37) nor by national centrality (β = -.04; B = -0.52, *p* = .54, 95% CI: -2.21, 1.19).

We also considered the possibility that associations with British national identity might be attenuated or confounded by the complexities of national identities among people in Scotland, Wales and Northern Ireland. Hence, we ran an alternative set of analyses focusing exclusively on participants residing in England. In these analyses, the association of national identity with mental well-being was reduced to marginal significance (*p* = .067) and the association with proximal helping was reduced to non-significance (*p* = .116), but the negative association with distal helping remained significant (*p* < .001). Thus, we found no evidence to suggest that the poorer outcomes associated with British national identification, compared to other identity categories, were attributable to anomalous results in Scotland, Wales or Northern Ireland.

# Study 2: Supplementary Information

## Historical Timeline and Data Corpus

In the UK and New Zealand, the first confirmed cases of COVID-19 were reported respectively on January 29^th^ and February 28^th^, the first cases of local transmission were confirmed respectively on February 28^th^ and March 5^th^, and the first COVID-related deaths were reported respectively on March 5^th^ and March 29^th^. We analysed the first 10 speeches and briefings regarding COVID-19 given by each country’s Prime Minister—Boris Johnson (UK) and Jacinda Ardern (New Zealand)—at the outset of the pandemic. These were given between March 9^th^ and 27^th^ for Johnson and between March 14^th^ and April 20^th^ for Ardern. At this initial stage of the crisis, both leaders urgently needed to mobilise their followers from no action to unprecedented action (e.g., lockdown measures). We also found that Boris Johnson became sick with COVID-19 after his first 10 speeches, providing a natural break in the historical sequence. Official transcripts, sourced from the respective governments’ websites, were of similar length (Ardern: ~10,500 words; Johnson: ~8,500 words).

## Qualitative Data Analysis

The qualitative analysis for Study 2 was conducted in four phases. Prior to beginning the analyses, the data and research questions were agreed between VV and ZJ. The main analysis was conducted by ZJ and EN, with additional input from VV.

**Phase 1.** ZJ independently analysed the two leaders’ rhetoric by iteratively appraising abductively the data (Locke et al., 2008). This author identified some theoretically relevant themes and discussed and refined these with EN.

**Phase 2.** After EN reviewed the speeches, the two authors settled on two very broad themes: *Leader Self-Positioning in Requests to Public*, and *Constructing the Group and Defining its Content, Norms and Interests*. Together the two authors started developing some NVivo codes within each theme, by iteratively exploring the leaders’ rhetoric vis-à-vis existing theories (Corbin & Strauss, 1990), which have now been captured in the literature review.

**Phase 3.** ZJ and EN independently coded the leaders’ speeches in NVivo and compared codes, iteratively, until they reached consensus around two second-order concepts per theme. For Theme I, the second-order concepts were: *Positioning Self and Audience* and *Framing Government Decisions*; for Theme II, they were: *Invoking Collective Resilience* and *Framing Protective Actions*. To illustrate these analytical concepts, ZJ and EN identified three representative quotes per second order concept and co-wrote an initial draft of the analysis. These quotes are reported partly in text (in the paper) and partly in Table S6, below.

**Phase 4.** The process of coding was explained to VV. This author checked the second-order concepts and quotes for consistency, discussing some possible refinements to the analysis with the initial two authors. All three authors worked collaboratively on a final draft of the analysis.

A final note is important: Of the four second-order concepts, we found that one subtheme—Invoking Collective Resilience by appealing to nationalistic and wartime rhetoric—was less novel (cf., Billig, 1995; Reicher & Hopkins, 2001). Due to restrictions on the word limit, we report this subtheme very succinctly in the paper, omitting to include one of the three quotes in text. This subtheme therefore appears with three quotes in Table S6.

## Theoretical Forestructure, Groundedness and Reflexive Validity

The goal of an in-depth qualitative analysis such as this is to generate novel insights through the researchers’ intensive engagement with the data. The analysis reported here was informed by the researchers’ prior expertise in leadership (e.g., Jaser, in press), social identity processes in extreme events (e.g., Ntontis et al., 2019), and identity construction (e.g., Vignoles, 2019). In the process of coding, we mitigated interpreters’ bias, and increased validity, by constantly comparing results between authors, and with theory (Stiles, 1993). Similar to previous studies of identity-based leadership, our first-order themes explore how leaders constructed themselves and their audiences (e.g., Haslam et al., 2020; Reicher et al., 2006). However, the deeper analysis of second-order concepts generated novel insights about the leaders’ framing of decisions as moral imperatives (in which they have a personal stake) versus technical requirements (in which they may not), and their constructions of protective actions as mutual helping or helping others. Thus, our theoretical forestructure was changed by the data, confirming that the analysis has reflexive validity (Stiles, 1993). Moreover, we illustrate our analysis with verbatim quotes from the two leaders’ speeches, with further quotations in Table S6, allowing readers to evaluate independently the grounding and persuasiveness of our interpretations (Levitt et al., 2017).

# Additional References

Becker, J. C., & Tausch, N. (2014). When group memberships are negative: The concept, measurement, and behavioral implications of psychological disidentification. *Self and Identity*, *13*(3), 294-321.

Billig, M. (1995). *Banal nationalism*. Sage.

Busby, D. M., & Yoshida, K. (2015). Challenges with online research for couples and families: Evaluating nonrespondents and the differential impact of incentives. *Journal of Child and Family Studies*, *24*(2), 505-513.

Corbin, J. M., & Strauss, A. (1990). Grounded theory research: Procedures, canons, and evaluative criteria. *Qualitative Sociology*, *13*(1), 3-21.

Eisinga, R., te Grotenhuis, M., & Pelzer, B. (2013). The reliability of a two-item scale: Pearson, Cronbach, or Spearman-Brown? *International Journal of Public Health, 58*, 637-642.

Institute of Race Relations (2020). *Ethnicity and religion statistics*. <https://irr.org.uk/research/statistics/ethnicity-and-religion/>

Jaser, Z. (Ed.) (in press). *The connecting leader: Serving concurrently as a leader and a follower.* Information Age Publishing.

Koc, Y. (2017). *Identity threat and coping strategies among highly stigmatised sexual and ethnic minorities*. Unpublished doctoral thesis, University of Sussex, UK.

Kroenke, K., Spitzer, R. L., Williams, J. B., & Löwe, B. (2009). An ultra-brief screening scale for anxiety and depression: the PHQ–4. *Psychosomatics*, *50*(6), 613-621.

Leach, C. W., Van Zomeren, M., Zebel, S., Vliek, M. L., Pennekamp, S. F., Doosje, B., ... & Spears, R. (2008). Group-level self-definition and self-investment: a hierarchical (multicomponent) model of in-group identification. *Journal of Personality and Social Psychology*, *95*, 144-165.

Levitt, H. M., Motulsky, S. L., Wertz, F. J., Morrow, S. L., & Ponterotto, J. G. (2017). Recommendations for designing and reviewing qualitative research in psychology: Promoting methodological integrity. *Qualitative Psychology*, *4*, 2-22.

Locke, K., Golden-Biddle, K., & Feldman, M. S. (2008). Making doubt generative: Rethinking the role of doubt in the research process. *Organization Science*, *19*(6), 907-918.

Ntontis, E., Drury, J., Amlôt, R., Rubin, G. J., & Williams, R. (2019). What lies beyond social capital? The role of social psychology in building community resilience to climate change. *Traumatology*. <https://doi.org/10.1037/trm0000221>

Office for National Statistics (2020). *Population estimates for the UK, England and Wales, Scotland and Northern Ireland: mid-2019*. <https://www.ons.gov.uk/peoplepopulationandcommunity/populationandmigration/populationestimates/bulletins/annualmidyearpopulationestimates/mid2019estimates#population-growth-in-england-wales-scotland-and-northern-ireland>

Podsakoff, P. M., MacKenzie, S. B., & Podsakoff, N. P. (2012). Sources of method bias in social science research and recommendations on how to control it. *Annual Review of Psychology, 63,* 539–569.

Rubin, G. J., Bakhshi, S., Amlôt, R., Fear, N., Potts, H. W., & Michie, S. (2014). The design of a survey questionnaire to measure perceptions and behaviour during an influenza pandemic: The Flu TElephone Survey Template (FluTEST). *Health Services and Delivery Research*, No. 2.41. <https://www.ncbi.nlm.nih.gov/books/NBK263566/>

Stewart-Brown, S. L., Platt, S., Tennant, A., Maheswaran, H., Parkinson, J., Weich, S., ... & Clarke, A. (2011). The Warwick-Edinburgh Mental Well-being Scale (WEMWBS): A valid and reliable tool for measuring mental well-being in diverse populations and projects. *J Epidemiology and Community Health*, *65*(Suppl 2), A38-A39.

Stiles, W. B. (1993). Quality control in qualitative research. *Clinical Psychology Review*, *13*(6), 593-618.

Tennant, R., Hiller, L., Fishwick, R., Platt, S., Joseph, S., Weich, S., ... & Stewart-Brown, S. (2007). The Warwick-Edinburgh mental well-being scale (WEMWBS): development and UK validation. *Health and Quality of life Outcomes*, *5*, 63.

Vignoles, V. L. (2019). Identity: Personal AND social. In K. Deaux & M. Snyder (Eds.), *Oxford handbook of personality and social psychology* (2nd ed., pp. 289–315). Oxford University Press.

Whitaker, C., Stevelink, S., & Fear, N. (2017). The use of Facebook in recruiting participants for health research purposes: a systematic review. *Journal of Medical Internet Research*, *19*(8), e290.

Table S1. *Descriptive statistics and rotated component loadings for items measuring protective and prosocial actions.*

| Items | N | % applicability | % likelihood | Rotated component loadings | | | |
| --- | --- | --- | --- | --- | --- | --- | --- |
|  |  |  |  | I | II | III | IV |
| ***Personal hygiene*** |  |  |  |  |  |  |  |
| Carried hand sanitising gel when you're out and about | 536 | 96% | 67% | **0.998** | 0.170 | 0.100 | -0.107 |
| Cleaned or disinfected things you might touch (doorknobs, surfaces) more often than usual | 559 | 100% | 74% | **0.994** | 0.055 | -0.039 | -0.173 |
| Used hand sanitising gel to clean your hands, more often than usual | 551 | 98% | 75% | **0.961** | 0.213 | 0.116 | -0.339 |
| Reduced the amount that you touched your eyes, nose, or mouth | 559 | 100% | 69% | **0.959** | 0.092 | -0.088 | 0.097 |
| Usually carried tissues with you when out and about | 534 | 95% | 60% | **0.922** | -0.083 | 0.005 | 0.307 |
| Usually used tissues when coughing or sneezing | 498 | 89% | 74% | **0.894** | 0.012 | -0.041 | 0.346 |
| Sneezed or coughed on your elbow | 524 | 94% | 76% | **0.711** | 0.019 | -0.067 | **0.614** |
| Washed your hands more frequently or thoroughly than usual | 559 | 100% | 96% | **0.571** | 0.046 | **-0.671** | **-0.442** |
| ***Proximal helping*** |  |  |  |  |  |  |  |
| Shopped for other households | 548 | 98% | 55% | 0.010 | **0.991** | -0.126 | -0.074 |
| Collected medication for someone on their behalf | 537 | 96% | 29% | 0.250 | **0.962** | 0.023 | 0.003 |
| Offered to shop for vulnerable people | 524 | 94% | 67% | 0.018 | **0.953** | -0.226 | 0.081 |
| Offered to help people with their computers to set up Skype, Facetime, etc. with their loved ones | 500 | 89% | 42% | 0.025 | **0.887** | -0.218 | 0.305 |
| Given up supplies to let someone vulnerable have them | 485 | 87% | 48% | 0.325 | **0.793** | 0.037 | **0.418** |
| ***Physical distancing*** |  |  |  |  |  |  |  |
| Stayed away from loved ones (family, partner, friends) to adhere to the rules of the lockdown | 551 | 98% | 96% | 0.009 | 0.050 | **-0.987** | -0.227 |
| Tried to avoid people who have the virus | 489 | 87% | 93% | 0.073 | 0.080 | **-0.982** | -0.139 |
| Used video calls to talk to loved ones (e.g. Skype, Facetime, etc.) instead of meeting in person | 552 | 99% | 91% | -0.308 | 0.273 | **-0.955** | -0.107 |
| Kept away from crowded places generally | 553 | 99% | 98% | 0.169 | -0.032 | **-0.944** | -0.280 |
| Reduced the amount that you go into shops | 552 | 99% | 95% | 0.319 | -0.059 | **-0.904** | -0.085 |
| Cancelled or postponed a social event such as meeting friends, eating out or going to a sports event | 528 | 94% | 94% | -0.001 | 0.343 | **-0.903** | 0.140 |
| Reduced or changed the way you use public transport | 367 | 66% | 90% | -0.128 | -0.066 | **-0.895** | **0.423** |
| Stayed at home at all times unless you were buying essentials, doing essential work, or exercising | 559 | 100% | 94% | 0.437 | -0.226 | **-0.801** | 0.088 |
| Kept one of your children out of school or nursery | 187 | 33% | 80% | -0.304 | 0.580 | **-0.792** | -0.015 |
| Reduced the amount you go to school, college, university or work | 452 | 81% | 83% | **-0.449** | -0.093 | **-0.792** | **0.523** |
| Stayed at least 2 metres at all times from people who are not part of your household | 553 | 99% | 88% | **0.440** | -0.060 | **-0.765** | 0.256 |
| Met with people outside your household for non- essential reasons | 559 | 100% | 12% | **-0.748** | 0.238 | **0.475** | -0.178 |
| Self-isolated | 548 | 98% | 60% | 0.062 | **-0.607** | **-0.457** | **0.667** |
| ***Distal helping*** |  |  |  |  |  |  |  |
| Signed a petition for housing rights during the pandemic | 538 | 96% | 22% | -0.113 | -0.103 | -0.173 | **0.984** |
| Donated to food banks | 549 | 98% | 24% | 0.217 | 0.264 | 0.236 | **0.893** |
| Given blood | 534 | 95% | 3% | -0.087 | 0.085 | **0.508** | **0.887** |
| Donated supplies/money to a homeless person | 533 | 95% | 25% | 0.125 | 0.342 | 0.310 | **0.869** |
| Offered to volunteer for the NHS | 522 | 93% | 11% | -0.260 | **0.418** | 0.054 | **0.867** |
| Donated money to charity or NHS | 555 | 99% | 55% | 0.285 | 0.254 | -0.291 | **0.788** |
| ***Excluded item*** |  |  |  |  |  |  |  |
| Followed a healthy diet or took vitamin supplements | 555 | 99% | 63% | **0.412** | **-0.423** | -0.171 | **0.765** |

*Note.* Total *N* = 560. % applicability is the percentage of participants who answered yes, no or not sure (rather than N/A) to an item. % likelihood is the estimated percentage of participants who had performed the behaviour (counting “not sure” as indicating 50% likelihood to have performed the behaviour). Rotated component loadings > .4 are shown in bold.

Table S2. *Means, standard deviations and zero-order correlations of main Study 1 measures.*

| Measure | N | Mean | (SD) |  | 1 | 2 | 3 | 4 |  | 5 | 6 | 7 | 8 |  | 9 | 10 | 11 | 12 |
| --- | --- | --- | --- | --- | --- | --- | --- | --- | --- | --- | --- | --- | --- | --- | --- | --- | --- | --- |
| 1. Personal hygiene | 560 | 73.87% | (23.20) |  | - |  |  |  |  |  |  |  |  |  |  |  |  |  |
| 1. Physical distancing | 560 | 88.96% | (11.87) |  | .28 | - |  |  |  |  |  |  |  |  |  |  |  |  |
| 1. Helping proximal others | 557 | 48.27% | (32.82) |  | .21 | .12 | - |  |  |  |  |  |  |  |  |  |  |  |
| 1. Helping distal others | 560 | 23.64% | (21.50) |  | .17 | .15 | .29 | - |  |  |  |  |  |  |  |  |  |  |
|  |  |  |  |  |  |  |  |  |  |  |  |  |  |  |  |  |  |  |
| 1. Mental well-being | 560 | 3.24 | (0.66) |  | -.06 | -.02 | .07 | -.02 |  | - |  |  |  |  |  |  |  |  |
| 1. Anxiety | 560 | 2.13 | (0.93) |  | .16 | .06 | .02 | .02 |  | -.61 | - |  |  |  |  |  |  |  |
| 1. Depression | 560 | 2.02 | (0.85) |  | .07 | .02 | -.05 | .01 |  | -.71 | .69 | - |  |  |  |  |  |  |
| 1. Perceived severity | 559 | 9.68 | (1.83) |  | .27 | .38 | .12 | .07 |  | -.04 | .09 | -.01 | - |  |  |  |  |  |
|  |  |  |  |  |  |  |  |  |  |  |  |  |  |  |  |  |  |  |
| 1. Family identification | 560 | 5.44 | (1.13) |  | .14 | .14 | .16 | .05 |  | .20 | -.18 | -.22 | .25 |  | - |  |  |  |
| 1. Local community identification | 560 | 4.51 | (1.11) |  | .10 | .10 | .17 | .18 |  | .20 | -.15 | -.24 | .16 |  | .33 | - |  |  |
| 1. National identification | 557 | 4.73 | (1.21) |  | .08 | .04 | .14 | -.07 |  | .15 | -.10 | -.10 | .13 |  | .23 | .08 | - |  |
| 1. Humanity identification | 557 | 5.16 | (0.99) |  | .04 | .10 | .10 | .14 |  | .26 | -.22 | -.32 | .17 |  | .34 | .41 | .09 | - |

Table S3. *Bivariate correlations with 95% bias-corrected accelerated confidence intervals (CI; 10000 requested bootstrap resamples) testing the associations between social identification and outcome measures.*

| Predictors |  | Behavioural outcomes | | | |  | | Psychological outcomes | | | |
| --- | --- | --- | --- | --- | --- | --- | --- | --- | --- | --- | --- |
|  |  | Personal hygiene | Physical distancing | Proximal helping | Distal helping |  | | Mental  well-being | Anxiety | Depressive symptoms | Perceived severity |
| Family identification | r | **.14***** | **.14**** | **.16***** | .05 | | **.21***** | | **-.18***** | **-.22***** | **.25***** |
| [95% CI] | | **[.06, .23]** | **[.04, .23]** | **[.08, .24]** | [-.03, .13] | | **[.13, .30]** | | **[-.26, -.09]** | **[-.31, -.14]** | **[.17, .33]** |
| Community identification | r | **.11*** | **.10*** | **.17***** | **.17***** | | **.19***** | | **-.14***** | **-.23***** | **.16***** |
| [95% CI] | | **[.02, .19]** | **[.02, .18]** | **[.08, .25]** | **[.09, .25]** | | **[.10, .27]** | | **[-.23, -.05]** | **[-.32, -.15]** | **[.09, .24]** |
| National identification | r | .07† | .04 | **.14***** | -.07† | | **.15***** | | **-.10*** | **-.10*** | **.13**** |
| [95% CI] | | [-.01, .16] | [-.05, .12] | **[.06, .22]** | [-.15, .01] | | **[.06, .24]** | | **[-.19, -.02]** | **[-.19, -.02]** | **[.04, .22]** |
| Humanity identification | r | .04 | **.10*** | **.10*** | **.14**** | | **.25***** | | **-.22***** | **-.32***** | **.17***** |
| [95% CI] | | [-.04, .12] | **[.01, .19]** | **[.01, .18]** | **[.06, .22]** | | **[.17, .34]** | | **[-.30, -.13]** | **[-.39, -.24]** | **[.08, .25]** |
|  | |  |  |  |  | |  | |  |  |  |

*Note*. Significant correlations are in bold.

****p* < .001, ** *p* < .01, * *p* < .05, † *p* < .10

Table S4. *Multiple regression parameters with 95% bias-corrected accelerated confidence intervals (CI; 10000 requested bootstrap resamples) testing the associations between social identification and behavioural outcome measures.*

| Predictors | Behavioural outcomes | | | | | | | | | | | | | | | | | | |
| --- | --- | --- | --- | --- | --- | --- | --- | --- | --- | --- | --- | --- | --- | --- | --- | --- | --- | --- | --- |
|  | Personal hygiene | | | |  | Physical distancing | | | |  | Proximal helping | | | |  | Distal helping | | | |
|  | B | | [95% CI] | |  | B | | [95% CI] | |  | B | | [95% CI] | |  | B | | [95% CI] | |
| *Model 1 [control]* |  | |  |  |  |  | |  |  |  |  | |  |  |  |  | |  |  |
| Intercept | **54.25** | ******* | **36.43** | **73.51** |  | **92.03** | ******* | **81.38** | **103.62** |  | **40.16** | ****** | **16.38** | **63.97** |  | 14.44 | † | -2.21 | 31.43 |
| Age | **0.30** | ******* | **0.16** | **0.44** |  | 0.05 |  | -0.03 | 0.14 |  | 0.07 |  | -0.12 | 0.26 |  | **0.17** | ***** | **0.04** | **0.30** |
| Gender (male) | **-8.51** | ****** | **-14.13** | **-2.90** |  | **-5.08** | ******* | **-8.00** | **-2.17** |  | **-11.98** | ****** | **-19.30** | **-4.45** |  | **-7.54** | ****** | **-12.17** | **-2.45** |
| Residence (Scotland) | 1.70 |  | -7.04 | 9.58 |  | -2.29 |  | -6.37 | 1.45 |  | 4.17 |  | -7.57 | 15.84 |  | -3.68 |  | -12.02 | 3.94 |
| Residence (Wales) | 0.59 |  | -12.13 | 12.05 |  | 2.37 |  | -1.29 | 5.63 |  | 11.28 |  | -3.37 | 25.35 |  | -1.67 |  | -11.12 | 6.80 |
| Residence (N. Ireland) | -1.20 |  | -21.33 | 23.57 |  | 0.06 |  | -7.22 | 8.52 |  | -16.61 |  | -34.93 | 1.16 |  | 8.82 |  | -5.07 | 27.77 |
| Working as normal | 2.16 |  | -2.57 | 7.19 |  | **-6.55** | ******* | **-8.97** | **-4.06** |  | 4.09 |  | -3.07 | 11.50 |  | -4.00 | **†** | -8.56 | 0.47 |
| Working remotely | -2.78 |  | -6.98 | 1.28 |  | 1.73 | † | -0.15 | 3.59 |  | 4.26 |  | -2.08 | 10.40 |  | 2.69 |  | -1.51 | 6.77 |
| Furloughed | 2.60 |  | -2.21 | 7.27 |  | **3.88** | ******* | **1.73** | **6.05** |  | 3.65 |  | -3.92 | 10.98 |  | 3.04 |  | -1.93 | 7.88 |
| Lost job | 5.13 |  | -9.24 | 17.84 |  | 0.14 |  | -5.75 | 5.68 |  | 0.98 |  | -18.61 | 21.74 |  | -2.09 |  | -15.03 | 12.42 |
| Other work impact | -1.52 |  | -6.26 | 3.17 |  | 2.03 | † | -0.11 | 4.14 |  | -3.79 |  | -11.07 | 3.28 |  | 1.86 |  | -3.19 | 6.98 |
| Personal symptoms | 2.09 |  | -0.35 | 4.37 |  | 0.28 |  | -1.14 | 1.60 |  | -0.32 |  | -4.12 | 3.61 |  | **3.14** | ***** | **0.74** | **5.71** |
| Others’ symptoms | 1.42 |  | -0.43 | 3.40 |  | 0.11 |  | -0.95 | 1.10 |  | **3.00** | ***** | **0.26** | **5.51** |  | 0.89 |  | -0.74 | 2.55 |
| Days into lockdown | 0.13 |  | -0.34 | 0.56 |  | -0.15 |  | -0.46 | 0.16 |  | 0.00 |  | -0.67 | 0.67 |  | -0.02 |  | -0.46 | 0.43 |
| Model 1 *R^2^* | **8.1%***** | | | |  | **11.9%***** | | | |  | **6.5%***** | | | |  | **6.1%**** | | | |
|  |  | |  |  |  |  | |  |  |  |  | |  |  |  |  | |  |  |
| *Model 2 [identification]* |  |  |  |  |  |  |  |  |  |  |  |  |  |  |  |  |  |  |  |
| Intercept | **45.53** | ******* | **23.54** | **69.45** |  | **81.81** | ******* | **70.27** | **93.70** |  | 3.71 |  | -26.48 | 33.08 |  | 3.74 |  | -16.22 | 23.44 |
| Age | **0.26** | ****** | **0.11** | **0.41** |  | 0.01 |  | -0.07 | 0.09 |  | -0.09 |  | -0.28 | 0.11 |  | **0.13** | ***** | **0.00** | **0.27** |
| Gender (male) | **-7.97** | ****** | **-13.57** | **-2.51** |  | **-4.72** | ****** | **-7.68** | **-1.68** |  | **-10.09** | ****** | **-17.52** | **-2.58** |  | **-8.35** | ******* | **-12.98** | **-3.28** |
| Residence (Scotland) | 1.42 |  | -7.71 | 9.54 |  | -2.21 |  | -6.37 | 1.54 |  | 4.88 |  | -6.30 | 16.06 |  | -4.90 |  | -13.32 | 2.81 |
| Residence (Wales) | -0.18 |  | -12.29 | 11.20 |  | 1.92 |  | -1.76 | 5.31 |  | 7.16 |  | -5.91 | 20.59 |  | -0.26 |  | -9.77 | 8.70 |
| Residence (N. Ireland) | -0.16 |  | -20.42 | 23.61 |  | 0.51 |  | -6.52 | 8.68 |  | -11.83 |  | -30.42 | 4.32 |  | 7.51 |  | -7.89 | 26.60 |
| Working as normal | 2.05 |  | -2.74 | 7.14 |  | **-6.81** | ******* | **-9.15** | **-4.40** |  | 3.12 |  | -4.03 | 10.40 |  | -2.82 |  | -7.25 | 1.60 |
| Working remotely | -2.95 |  | -7.22 | 1.16 |  | 1.60 | † | -0.23 | 3.40 |  | 3.73 |  | -2.64 | 9.94 |  | 1.31 |  | -2.73 | 5.37 |
| Furloughed | 2.85 |  | -1.90 | 7.60 |  | **4.05** | ******* | **1.91** | **6.16** |  | 4.75 |  | -2.72 | 11.89 |  | 4.21 | † | -0.55 | 8.81 |
| Lost job | 4.80 |  | -9.80 | 17.47 |  | 0.43 |  | -5.21 | 5.51 |  | 1.38 |  | -17.86 | 21.80 |  | -3.19 |  | -15.86 | 10.51 |
| Other work impact | -1.33 |  | -6.14 | 3.53 |  | 1.91 | † | -0.17 | 4.02 |  | -3.73 |  | -10.99 | 3.42 |  | 2.08 |  | -2.73 | 6.96 |
| Personal symptoms | 2.14 | † | -0.30 | 4.48 |  | 0.32 |  | -1.08 | 1.64 |  | -0.17 |  | -3.84 | 3.72 |  | **3.21** | ****** | **0.86** | **5.77** |
| Others’ symptoms | 1.29 |  | -0.55 | 3.26 |  | 0.02 |  | -1.02 | 0.96 |  | 2.42 | † | -0.31 | 4.92 |  | 0.85 |  | -0.78 | 2.51 |
| Days into lockdown | 0.14 |  | -0.33 | 0.60 |  | -0.16 |  | -0.48 | 0.15 |  | -0.06 |  | -0.72 | 0.63 |  | 0.06 |  | -0.38 | 0.51 |
| Family identification | 1.52 |  | -0.51 | 3.43 |  | **1.15** | ***** | **0.12** | **2.25** |  | 2.33 |  | -0.54 | 5.05 |  | -0.66 |  | -2.30 | 0.93 |
| Community identification | 1.40 |  | -0.52 | 3.45 |  | 0.33 |  | -0.62 | 1.32 |  | **3.50** | ***** | **0.68** | **6.19** |  | **2.83** | ****** | **1.18** | **4.44** |
| National identification | 0.03 |  | -1.66 | 1.67 |  | 0.27 |  | -0.52 | 1.06 |  | **2.76** | ***** | **0.37** | **5.13** |  | **-2.04** | ****** | **-3.44** | **-0.72** |
| Humanity identification | -0.88 |  | -3.00 | 1.18 |  | 0.70 |  | -0.49 | 1.84 |  | 1.14 |  | -2.04 | 4.38 |  | 1.81 | **†** | -0.21 | 3.79 |
| Model 2 Δ*R^2^* | 1.0% | | | |  | **2.3%**** | | | |  | **4.2%***** | | | |  | **4.1%***** | | | |

*Note*. Significant parameters are in bold. Gender was coded (0 = female; 0.5 = non-binary/unspecified; 1 = male). Nation of residence was contrast coded (-1 = England; 1 = target nation; 0 otherwise). Working impacts were contrast coded (-1 = not applicable; 1 = target category; 0 = otherwise).

****p* < .001, ** *p* < .01, * *p* < .05, † *p* < .10

Table S5. *Multiple regression parameters with 95% bias-corrected accelerated confidence intervals (CI; 10000 requested bootstrap resamples) testing the associations between social identification and psychological outcome measures.*

| Predictors | Psychological outcomes | | | | | | | | | | | | | | | | | | |
| --- | --- | --- | --- | --- | --- | --- | --- | --- | --- | --- | --- | --- | --- | --- | --- | --- | --- | --- | --- |
|  | Mental well-being | | | |  | Anxiety | | | |  | Depressive symptoms | | | |  | Perceived severity | | | |
|  | B | | [95% CI] | |  | B | | [95% CI] | |  | B | | [95% CI] | |  | B | | [95% CI] | |
| *Model 1 [control]* |  | |  |  |  |  | |  |  |  |  | |  |  |  |  | |  |  |
| Intercept | **2.91** | ******* | **2.41** | **3.43** |  | **2.48** | ******* | **1.75** | **3.16** |  | **2.44** | ******* | **1.83** | **3.02** |  | **8.94** | ******* | **7.43** | **10.49** |
| Age | **0.01** | ******* | **0.01** | **0.01** |  | **-0.01** | ******* | **-0.02** | **-0.01** |  | **-0.01** | ******* | **-0.02** | **-0.01** |  | **0.03** | ******* | **0.02** | **0.04** |
| Gender (male) | 0.02 |  | -0.11 | 0.15 |  | **-0.23** | ***** | **-0.43** | **-0.02** |  | -0.07 |  | -0.25 | 0.12 |  | **-0.72** | ****** | **-1.17** | **-0.28** |
| Residence (Scotland) | -0.06 |  | -0.33 | 0.19 |  | -0.05 |  | -0.36 | 0.28 |  | 0.08 |  | -0.19 | 0.37 |  | -0.06 |  | -0.66 | 0.53 |
| Residence (Wales) | 0.09 |  | -0.21 | 0.38 |  | -0.20 |  | -0.57 | 0.20 |  | -0.21 |  | -0.51 | 0.12 |  | 0.22 |  | -0.57 | 0.98 |
| Residence (N. Ireland) | 0.09 |  | -0.40 | 0.66 |  | 0.17 |  | -0.40 | 0.68 |  | -0.02 |  | -0.55 | 0.46 |  | -0.26 |  | -1.66 | 1.10 |
| Working as normal | **0.27** | ******* | **0.13** | **0.41** |  | **-0.18** | ***** | **-0.36** | **0.00** |  | **-0.24** | ****** | **-0.42** | **-0.07** |  | 0.26 |  | -0.14 | 0.66 |
| Working remotely | **0.14** | ***** | **0.02** | **0.27** |  | -0.12 |  | -0.29 | 0.03 |  | **-0.20** | ***** | **-0.36** | **-0.05** |  | 0.16 |  | -0.16 | 0.50 |
| Furloughed | -0.08 |  | -0.24 | 0.09 |  | -0.18 | † | -0.38 | 0.02 |  | 0.08 |  | -0.13 | 0.28 |  | 0.29 |  | -0.13 | 0.71 |
| Lost job | -0.30 |  | -0.75 | 0.12 |  | **0.63** | ****** | **0.13** | **1.11** |  | 0.45 | † | -0.12 | 1.01 |  | -0.83 |  | -2.09 | 0.32 |
| Other work impact | 0.01 |  | -0.12 | 0.15 |  | -0.02 |  | -0.21 | 0.17 |  | -0.12 |  | -0.31 | 0.07 |  | 0.30 | † | -0.06 | 0.65 |
| Personal symptoms | -0.04 |  | -0.12 | 0.03 |  | **0.12** | ***** | **0.01** | **0.23** |  | 0.08 | † | -0.02 | 0.19 |  | **-0.22** | ***** | **-0.44** | **0.00** |
| Others’ symptoms | -0.01 |  | -0.06 | 0.04 |  | 0.07 | † | 0.00 | 0.14 |  | 0.02 |  | -0.04 | 0.09 |  | **0.20** | ****** | **0.06** | **0.32** |
| Days into lockdown | 0.00 |  | -0.01 | 0.01 |  | 0.00 |  | -0.02 | 0.02 |  | 0.00 |  | -0.02 | 0.01 |  | -0.02 |  | -0.06 | 0.02 |
| Model 1 *R^2^* | **9.0%***** | | | |  | **8.3%***** | | | |  | **8.6%***** | | | |  | **10.8%***** | | | |
|  |  | |  |  |  |  | |  |  |  |  | |  |  |  |  | |  |  |
| *Model 2 [identification]* |  |  |  |  |  |  |  |  |  |  |  |  |  |  |  |  |  |  |  |
| Intercept | **1.99** | ******* | **1.42** | **2.56** |  | **3.60** | ******* | **2.76** | **4.39** |  | **3.83** | ******* | **3.13** | **4.51** |  | **6.95** | ******* | **5.25** | **8.79** |
| Age | **0.01** | ****** | **0.00** | **0.01** |  | **-0.01** | ****** | **-0.01** | **0.00** |  | **-0.01** | ****** | **-0.01** | **0.00** |  | **0.02** | ******* | **0.01** | **0.03** |
| Gender (male) | 0.05 |  | -0.08 | 0.18 |  | **-0.26** | ****** | **-0.45** | **-0.06** |  | -0.09 |  | -0.26 | 0.10 |  | **-0.65** | ****** | **-1.10** | **-0.20** |
| Residence (Scotland) | -0.04 |  | -0.32 | 0.22 |  | -0.07 |  | -0.39 | 0.27 |  | 0.07 |  | -0.21 | 0.37 |  | -0.05 |  | -0.64 | 0.52 |
| Residence (Wales) | 0.03 |  | -0.28 | 0.32 |  | -0.14 |  | -0.53 | 0.27 |  | -0.16 |  | -0.46 | 0.17 |  | 0.14 |  | -0.60 | 0.87 |
| Residence (N. Ireland) | 0.15 |  | -0.38 | 0.78 |  | 0.11 |  | -0.52 | 0.67 |  | -0.06 |  | -0.68 | 0.49 |  | -0.18 |  | -1.56 | 1.14 |
| Working as normal | **0.25** | ******* | **0.11** | **0.38** |  | -0.16 | † | -0.34 | 0.03 |  | **-0.23** | ****** | **-0.40** | **-0.05** |  | 0.21 |  | -0.16 | 0.59 |
| Working remotely | **0.12** | ***** | **0.00** | **0.25** |  | -0.10 |  | -0.26 | 0.05 |  | **-0.16** | ***** | **-0.31** | **-0.01** |  | 0.14 |  | -0.17 | 0.46 |
| Furloughed | -0.06 |  | -0.21 | 0.10 |  | -0.20 | † | -0.41 | 0.00 |  | 0.04 |  | -0.16 | 0.23 |  | 0.33 |  | -0.08 | 0.72 |
| Lost job | -0.26 |  | -0.69 | 0.15 |  | **0.58** | ****** | **0.09** | **1.06** |  | 0.40 |  | -0.15 | 0.94 |  | -0.78 |  | -1.96 | 0.28 |
| Other work impact | 0.00 |  | -0.13 | 0.13 |  | -0.01 |  | -0.19 | 0.18 |  | -0.10 |  | -0.28 | 0.09 |  | 0.27 |  | -0.06 | 0.60 |
| Personal symptoms | -0.04 |  | -0.11 | 0.03 |  | **0.12** | ***** | **0.01** | **0.22** |  | 0.08 |  | -0.02 | 0.18 |  | **-0.21** | ***** | **-0.42** | **0.00** |
| Others’ symptoms | -0.02 |  | -0.07 | 0.03 |  | **0.08** | ***** | **0.01** | **0.15** |  | 0.04 |  | -0.03 | 0.10 |  | **0.18** | ***** | **0.04** | **0.30** |
| Days into lockdown | 0.00 |  | -0.01 | 0.01 |  | 0.00 |  | -0.02 | 0.02 |  | 0.00 |  | -0.02 | 0.01 |  | -0.02 |  | -0.06 | 0.02 |
| Family identification | 0.04 |  | -0.02 | 0.10 |  | -0.06 |  | -0.15 | 0.02 |  | -0.06 |  | -0.13 | 0.02 |  | **0.25** | ****** | **0.10** | **0.39** |
| Community identification | 0.04 |  | -0.02 | 0.10 |  | -0.04 |  | -0.12 | 0.05 |  | -0.07 | † | -0.14 | 0.00 |  | 0.07 |  | -0.06 | 0.21 |
| National identification | **0.05** | ***** | **0.00** | **0.10** |  | -0.05 |  | -0.12 | 0.02 |  | -0.03 |  | -0.09 | 0.03 |  | 0.04 |  | -0.09 | 0.17 |
| Humanity identification | **0.10** | ****** | **0.04** | **0.17** |  | **-0.13** | ****** | **-0.22** | **-0.04** |  | **-0.18** | ******* | **-0.26** | **-0.10** |  | 0.11 |  | -0.06 | 0.27 |
| Model 2 Δ*R^2^* | **5.9%***** | | | |  | **4.3%***** | | | |  | **8.5%***** | | | |  | **3.9%***** | | | |

*Note*. Significant parameters are in bold. Gender was coded (0 = female; 0.5 = non-binary/unspecified; 1 = male). Nation of residence was contrast coded (-1 = England; 1 = target nation; 0 otherwise). Working impacts were contrast coded (-1 = not applicable; 1 = target category; 0 = otherwise).

****p* < .001, ** *p* < .01, * *p* < .05, † *p* < .10

Table S6. *Additional quotations illustrating each discursive theme.*

| **Theme 1:** Leader Self-Positioning in Requests for Action | |  |
| --- | --- | --- |
| **Ardern** | **Johnson** |  |
| **Positioning Self and Audience** | |  |
| We need as a nation to prepare and manage the spread of COVID-19. | But I want to stress something that is very important in the wake of what we’re saying this afternoon – I urge people, who think in view of what we’re saying about their potential symptoms that they should stay at home. |  |
| If we want to make sure that we are a health success story and ensure our economy can start to operate again without the virus taking off, we need to get the next phase right. | I also want at this stage to speak directly to older people. Because this disease is particularly dangerous for you, for older people, even though the vast majority this will be a mild to moderate illness, I know that many people will be very worried. |  |
| **Framing Government Decisions** | |  |
| We have two choices as a nation. One is to let COVID-19 roll on, and brace. The second is to go hard on measures to keep it out and stamp it out - not because we can stop a global pandemic from reaching us, but because it is in our power to slow it down. I make no apology for choosing the second path. New Zealanders public health comes first. | And even if you don’t have symptoms and if no one in your household has symptoms, there is more that we need you to do now. |  |
| I do not underestimate what I am asking New Zealanders to do. It’s huge. And I know it will feel daunting. But I wanted to share with you the stark choice we face. […] The worst-case scenario is simply intolerable. It would represent the greatest loss of New Zealanders’ lives in our country’s history. | But the sad things is that today for now, at least physically, we need to keep people apart. |  |
| **Theme II:**  Constructing the National Group and Defining its Content, Norms and Interests | |  |
| **Ardern** | **Johnson** |  |
| **Invoking Collective Resilience** | |  |
| And so, this weekend, ANZAC weekend, enjoy the company of your bubble, stay local, and reflect on the amazing sacrifice of our forebears. Decades ago, they came together in the most testing of circumstances half a world away and helped forge who we are today. It was a very different battle than the one we are in now, but the character of who we are as a country remains exactly the same. So please, stay strong, stay home, be kind. And let’s finish what we started. | But in this fight we can be in no doubt that each and every one of us is directly enlisted. Each and every one of us is now obliged to join together. To halt the spread of this disease. To protect our NHS and to save many many thousands of lives. And I know that as they have in the past so many times. The people of this country will rise to that challenge. And we will come through it stronger than ever. |  |
| For now, I ask that New Zealand does what we do so well. We are a country that is creative, practical, and community minded. We may not have experienced anything like this in our lifetimes, but we know how to rally and we know how to look after one another, and right now what could be more important than that. So thank you for all that you’re about to do. | There is no hiding from the fact that the coronavirus outbreak will present significant challenges for the UK, just as it does in other countries. But if we continue to look out for one another, to pull together in a united and national effort, I have no doubt that we can and will rise to that challenge. |  |
| These measures, while disruptive, are needed to make the space we need as a nation to prepare and manage the spread of COVID-19. | We will get through this, this country will get through this epidemic, just as it has got through many tougher experiences before if we look out for each other and commit wholeheartedly to a full national effort. |  |
| **Framing Protective Actions** | |  |
| If you have any questions about what you can or can’t do, apply a simple principle. Act like you have COVID-19. Every move you then make is a risk to someone else. That is how we must all collectively think. That’s why the joy of physically visiting other family, children, grandchildren, friends, neighbours is on hold. Because we’re all now putting each other first. And that is what we as a nation do so well. | From tomorrow, if you have coronavirus symptoms, however mild – either a new continuous cough or a high temperature – then you should stay at home for at least 7 days to protect others and help slow the spread of the disease. |  |
| Finally, we are a tough resilient people. We have been here before. But our journey will depend on how we work together. We are taking every measure we need as a government, and we ask that you do to. We all have a role to play. Look out for your neighbour, look out for your family. Look out for your friends | So, to relieve the pressure on the London health system and to slow the spread in London, it’s important that Londoners now pay special attention to what we are saying about avoiding non-essential contact, and to take particularly seriously the advice about working from home, and avoiding confined spaces such as pubs and restaurants |  |

1. Another single-item rating, “How much have you altered your lifestyle because of the pandemic?”, was not used in our analyses, because the protective and prosocial behaviours measures gave a more detailed picture of lifestyle alterations. [↑](#footnote-ref-1)
2. Two further items asked whether participants had “bought more [groceries / medicines] than you normally would”. The final section explored reasons for doing so among those who responded yes. These actions are not straightforwardly protective or pro-/anti-social. We plan to write about them elsewhere, and we did not include them in the current analyses. [↑](#footnote-ref-2)
